# Reducing Uncertainty in the American Community Survey through Data-Driven Regionalization

**DOI:** 10.1371/journal.pone.0115626

**Published:** 2015-02-27

**Authors:** Seth E. Spielman, David C. Folch

**Affiliations:** 1 Geography Department and Institute of Behavioral Science, University of Colorado at Boulder, Boulder, Colorado, USA; 2 Department of Geography, Florida State University, Tallahassee, Florida, USA; Universidad Veracruzana, MEXICO

## Abstract

The American Community Survey (ACS) is the largest survey of US households and is the principal source for neighborhood scale information about the US population and economy. The ACS is used to allocate billions in federal spending and is a critical input to social scientific research in the US. However, estimates from the ACS can be highly unreliable. For example, in over 72% of census tracts, the estimated number of children under 5 in poverty has a margin of error greater than the estimate. Uncertainty of this magnitude complicates the use of social data in policy making, research, and governance. This article presents a heuristic spatial optimization algorithm that is capable of reducing the margins of error in survey data via the creation of new composite geographies, a process called regionalization. Regionalization is a complex combinatorial problem. Here rather than focusing on the technical aspects of regionalization we demonstrate how to use a purpose built open source regionalization algorithm to process survey data in order to reduce the margins of error to a user-specified threshold.

## Introduction

In 2010 the American Community Survey (ACS) replaced the long form of the decennial census as the principal source for geographically detailed information about the population and economy of the United States. The ACS produces estimates for thousands of variables at a variety of geographic scales, the smallest of which (the block group) divides the US like a jigsaw puzzle into 217,740 pieces. The ACS releases estimates annually; however, for smaller areas these annual estimates are based on 3 or 5 years of data collection. This increase in frequency comes at a cost, the ACS data are terribly imprecise. For some policy-relevant variables, like the number of children in poverty, the estimates are almost unusable—in the 2007–2011 ACS, of the 56,204 tracts for which a poverty estimate for children under 5 was available, 40,941 (72.8%) had a margin of error greater than the estimate. For example, the ACS indicates that Census Tract 196 in Brooklyn, New York has 169 children under 5 in poverty ± 174 children, suggesting that somewhere between 0 and 343 children in the area live in poverty.

At the census tract scale, the margins of error on ACS data are on average 75 percent larger than those of the corresponding decennial long form estimate [1]. The imprecision in the ACS is especially vexing because the survey is used to allocate nearly $450 billion in federal spending each year [2]. For example, the US Treasury Department’s New Markets Tax Credit (NMTC) provides a federal tax credit for investment in low-income communities. Since its inception in 2000 the NMTC has distributed over $36 billion in tax credits; unfortunately, the census tracts targeted by this program are especially ill-served by the ACS. Spielman, Folch, and Nagle [3] show that there is a strong association between tract-level median household income and data quality. The practical implication is that some places which arguably should qualify for public assistance are disqualified and vice versa: imprecision in public data has real social implications. It was well understood, before the adoption of the ACS, that the ACS would have higher margins of error than earlier decennial censuses. However, the difference in quality between the ACS and the decennial long form has far exceeded initial expectations. The particular reasons for this decline in data quality are complex and are discussed in detail elsewhere [3]. This paper focuses on a way to fix the data, that is, to reduce the margins of error in the ACS data to some user-specified quality threshold.

The method presented here is explicitly spatial: it reengineers census geography by combining tracts (or block groups) into larger “regions.” These regions, because they have a larger effective sample size, have a smaller margin of error. The process of building regions is computationally complex and fraught with conceptual (and practical) challenges. The algorithm that we present an overview of here has been previously described in the technical literature [4], the aim in this article is to illustrate how spatial optimization procedures can be used to improve the usability of small area estimates from the ACS (or any other survey). In the balance of this paper, we explain these challenges, present the region-building algorithm, and provide empirical results demonstrating the algorithm’s utility across a broad range of variables and geographic locations. The algorithm is open source and freely available at (https://github.com/geoss/ACS_Regionalization).

## Existing Strategies to Reduce the MOE in Survey Data

As the name suggests, the ACS is a survey. It aims to build population-level estimates based on information from a sample of the US population. The “populations” for which the ACS produces estimates are geographically defined and range in size from approximately 1500 people (block groups) to administrative units such as cities, counties, states, and the nation. The estimates for any given geographic area are created via a combination of data (completed questionnaires) and statistical methods (weighting and variance estimation). In 2012 3,539,552 households were contacted by the ACS, resulting in 2,375,715 completed surveys (a 67% response rate). The number of completed surveys seems substantial until one considers the number of geographic zones for which estimates are produced. In 2012, the most recent year for which data were available, this response rate translates into 32 responses per census tract and 11 responses per block group on average per year. At the tract and block group scale these data are pooled into multiyear estimates, giving an average of 135 (median 124) completed surveys per tract over the 5-year period from 2007 through 2011. However, the ACS produces over 1400 tables of estimates per tract making this average of 135 responses seem woefully inadequate. The ACS estimates describe geographically bounded populations, so the number of completed surveys within any given geographic area is largely a function of the population and the response rate. As one’s geographic zone of interest grows in size, the number of completed surveys increases. Zones with larger numbers of completed surveys have high-quality estimates. Thus for larger geographic scales like large counties and cities the ACS estimates are excellent and provide high-quality annual data, but for small areas like tracts and block groups the estimates are poor.

The US Census Bureau publishes margins of error (MOE) to accompany each estimated variable in the ACS. The published margins of error reflect a 90 percent confidence interval, a range of values that is overwhelmingly likely to contain the true population-level value for a given variable. These MOEs are published on the same scale as the variable—that is, the margin of error on an income variable is expressed in dollars and the margin of error on a count of people is expressed as number of people. This makes it difficult to directly compare the amount of uncertainty in a variable on a dollar scale with one on a count scale. For this reason we use a statistic called the coefficient of variation (CV), which is calculated as $CV_{ij}=\frac{MOE_{ij}/1.645}{ESTIMATE_{ij}}$ where *i* and *j* index areal units and variables, respectively. The CV is an imperfect but useful statistic because it gives a standardized measure of uncertainty that can be interpreted as the share of the estimate that the error represents—higher CV implies greater uncertainty. There is no CV level that is universally accepted as “too high,” but a comprehensive report on the ACS [5] describes a range of 0.10 to 0.12 as a “reasonable standard of precision for an estimate” (p. 64).

Although uncertainty in the ACS can be high, data users often have few, if any, alternatives; so researchers, planners, and policymakers must proceed using the currently available data. The US Census Bureau (USCB) offers two strategies for data users confronting high-uncertainty data: “while it is true that estimates with high CVs have important limitations, they can still be valuable as building blocks to develop estimates for higher levels of aggregation. Combining estimates across geographic areas or collapsing characteristic detail can improve the reliability of those estimates as evidenced by reductions in the CVs” (p. A-13). The first strategy, “collapsing detail,” and the second strategy, “combining geographic areas,” work by effectively increasing the sample size supporting a given estimate. If, for example, the CV on income for African-Americans in a census tract is too high, one could “collapse” detail by considering income for all residents of the tract (as opposed to just the subset of people who identify as African-American). However, this strategy is not viable for all variables. For example, the 2007–2011 ACS estimates of the number of people living in poverty show that in 3835 tracts the MOE is greater than the estimate, and in 35,737 tracts (approximately 50% of all tracts) the MOE is 50% or more of the estimate. While the ACS poverty estimates are very poor, they cannot be collapsed because no coarser level of detail is available.

**Fig 1:**
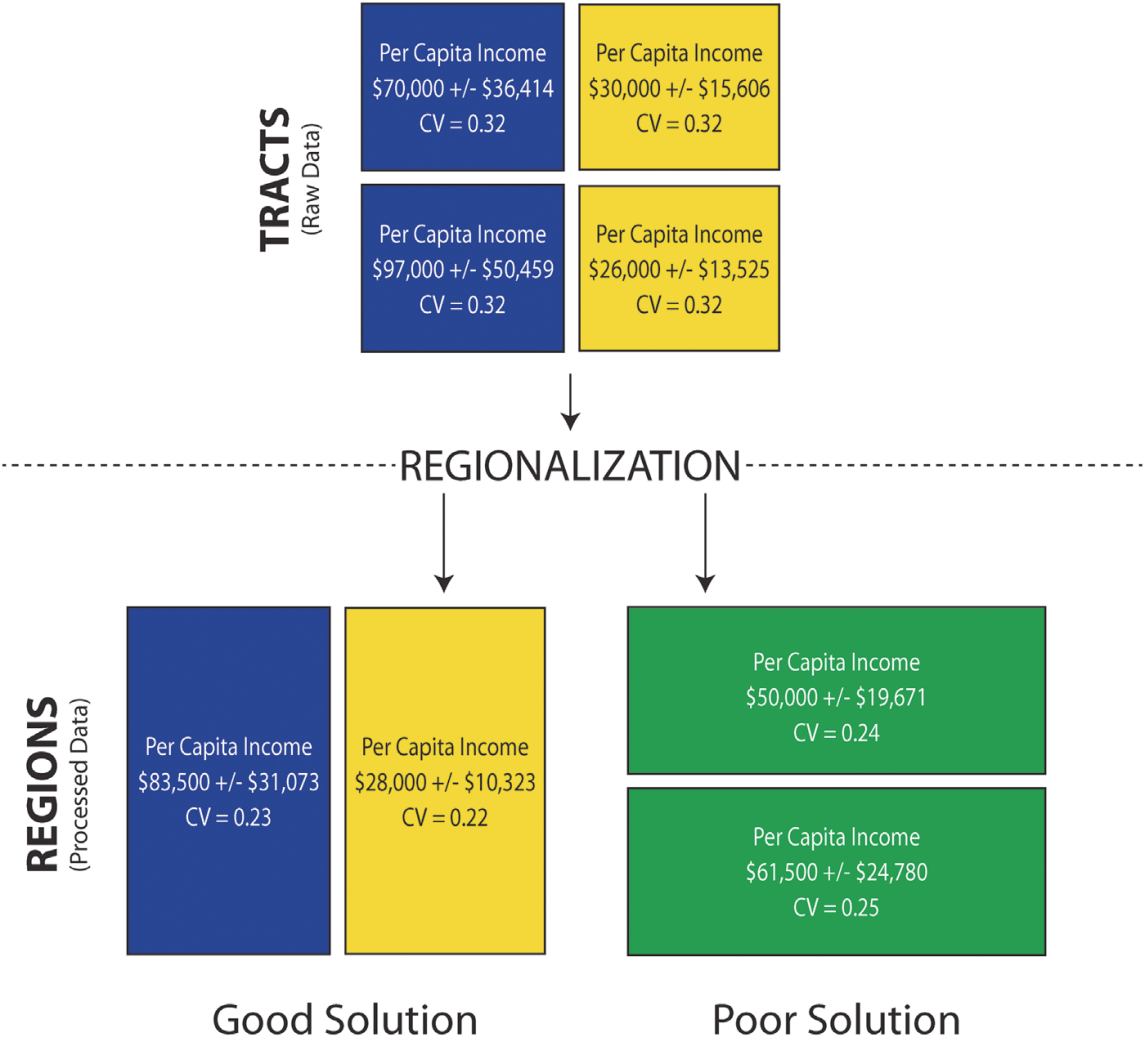
An illustration of “combining geographic areas” strategy.

The second strategy, “combining geographic areas” together into a “region,” works via a similar mechanism (boosting the number of completed surveys supporting an estimate): a group of census tracts will contain more completed surveys than a single tract, and thus will usually yield a reduced margin of error. This grouping strategy allows users to maintain attribute detail while achieving higher-quality estimates. This procedure is illustrated in Fig. 1. In the figure the squares represent census tracts and the rectangles show regions (combinations of two tracts). The color of the unit corresponds to the estimated per capita income, with blue representing high income and yellow representing low income. Per capita income simply divides aggregate income by the population. Each of the input tracts has an estimated population of 5000 ± 822 people (i.e., a CV of 0.1); aggregate income varies by tract but has a constant CV of 0.3.

As Fig. 1 shows, it is possible to significantly alter the geographic distribution of a variable via aggregation. That is, one can induce geographic patterns in the aggregate data that do not exist in the input data. For example, in the lower right of Fig. 1 a high-income neighborhood is combined with a low-income neighborhood, and while this reduces the margin of error it creates a green middle income neighborhood type that did not exist in the input data. A map can be “broken” by aggregations that mix dissimilar types of neighborhoods, thus creating new types of regions. In contrast the regions in the lower left maintain the same pattern as the original tract map. It has long been known that such aggregation effects can have a profound impact on analytical outcomes [6, 7].

The Census Bureau’s recommendation to combine geographic areas does not include a framework for solution quality, nor does it include a warning to users about the analytical implications of modifying areal units. However, it is clear that naively applied, the geographic aggregation strategy carries a real risk of generating spurious or at least questionable analytical results. The problem is compounded when one considers a multivariate case, because an aggregation that preserves patterns in one variable may “break” patterns in others. Complicating matters even more, if one expanded the four tracts in Fig. 1 to an entire metropolitan area that contains thousands of tracts, there would be millions (or more) of possible aggregations. A final wrinkle is that within metropolitan areas there can be substantial tract-to-tract and estimate-to-estimate variability in the quality of data [8]. A particular attribute will not have a constant CV in all census tracts (see Table 3)—some tracts may have good poverty estimates whereas other nearby tracts may not. A single tract may have a good poverty estimate but a poor income estimate. Thus it is unnecessary to apply a “collapsing detail” or “aggregating geography” strategy for every census tract. When applied naively to large areas, the aggregation strategies recommended by USCB to reduce the MOE will have a tendency to over-correct the problem. Since some input geographies will have reliable estimates across all variables of interest, these areas should not be combined with other tracts, because doing so would result in an unnecessary loss of geographic detail.

This article presents a multivariate algorithm for finding the “best” possible combination of tracts into new regions. The algorithm accepts a variety of inputs from the user, including a list of variables and a data quality threshold (CV). Given a large multivariate map of census geographies, it will enumerate a representative subset of the millions of possible combinations of tracts into regions. The algorithm employs an optimization procedure to get all variables over the user-specified quality threshold; for example, all variables must have a CV of less than 0.10. The algorithm attempts to minimize the amount of aggregation and maximize the quality of the output regions; it will group tracts together only when grouping is necessary, and avoids “poor” solutions (as in the lower right of Fig. 1) through an objective function that penalizes intraregion heterogeneity. We view this process as both an art and a science and thus provide both quantitative and visual procedures for assessing the quality of the algorithm’s solutions. Using this algorithm requires one to sacrifice geographic detail for attribute precision; however, as we show in subsequent sections, the magnitude of this trade-off is controlled by the user.

### Regionalization

Most Americans conceptualize “the South” or “New England” as regions. Montello [9] describes a region as a geographic category whose defining characteristic is that the entities it contains are in some way similar to each other and differentiated from entities in other categories. The process of regionalization (the identification of regions) is akin to drawing lines on a map that delineate the spatial extent and thus the membership of a region. In the case of New England this would mean grouping states or counties by circumscribing them within some boundary. Experts or residents who agree on the existence of a region will often differ on the exact boundaries that define it [10].

Regionalization is a general term that covers procedures in which *n* areas, such as census tracts, are grouped into *p* regions. The concept is similar to clustering, in which *n* observations are grouped into *p* “clusters” on the basis of similarity. Regionalization simply adds a spatial contiguity constraint to clustering algorithms, meaning that a region is a set of census tracts each of which touches at least one other member of the region. The *p* regions therefore cover the same territory as the *n* areas, but do so using fewer spatial units. For almost any real-world problem, there are far more potential groupings of areas into regions than can be tested to find the optimal solution; therefore heuristic algorithms must be employed to search the solution space intelligently. The heuristic algorithm described below identifies geographically contiguous clusters of tracts that are as homogeneous as possible across a user-specified set of attributes.

Census tracts are themselves often seen as substantively meaningful regions that group together residents into “neighborhoods” [11–16]. Given the substantive meaning ascribed to census tracts, and their widespread use, the Census Bureau decided to maintain the decennial geographic system of block groups and tracts for the ACS. Our algorithm does not discard the old system of census tracts but builds new regions based upon combinations of existing areas. This requires one to abandon those areas in favor of a new geography. While many users of census data are attached to tracts and see them as substantive units of analysis, we believe that such attachments are unwise given the quality of the ACS data. Even if census tracts are substantively important geographies that structure urban space, for many variables the data quality is so poor that it becomes impossible to differentiate areas on important characteristics like wealth, race, ethnicity, etc.

### Computing Regions with a User Specified Uncertainty

The computational regionalization algorithm developed here has three goals:

- Reduce the margin of error on input variables to meet or exceed a user specified threshold.
- Avoid grouping dissimilar areas together, i.e., do not break the pattern on the map.
- Group together as few tracts as necessary to meet user specified data quality thresholds.

To achieve the first goal, we require that every attribute in every region has a CV below a user-specified threshold. These thresholds can be global, so that all variables meet or exceed a user specified threshold *c* (i.e., *CV* ≤ *c*), or variable specific, so that for a set of *J* variables a user specifies a 1 × *J* vector of CV or MOE targets. In addition the user can specify a maximum, a minimum, or a range for the population of output regions. The second goal is achieved through the use of an objective function that aims to minimize intraregion heterogeneity. The objective function is simply the sum of the squared deviations (SSD) from the mean of the region for each variable:

$$\begin{array}{c} SSD=\sum_{k=1}^{p}\sum_{i\in k}\sum_{j=1}^{J}{\left(a_{ij}-{\bar{a}}_{ik}\right)}^{2}. \end{array}$$ (1)

There is some debate in the literature about objective functions for regionalization. Martin, Nolan, and Tranmer [17] have argued that minimizing the *intra*region heterogeneity as in equation 1 does not necessarily maximize the *inter*region heterogeneity. That is, the objective of regionalization should be to ensure that one creates internally homogeneous regions that are strongly differentiated from other regions. This approach, however, requires an arbitrary decision on how to weight inter- and intraregion composition.

The third goal is accomplished by maximizing the number of regions created from the input map of tracts, subject to user-specified constraints. By maximizing the number of output regions, we minimize aggregation.

We have adapted the max-*p* regionalization algorithm [18] to achieve these goals. The max-*p* algorithm operates in two phases. The first phase proceeds by selecting a census tract at random from all the tracts, and designates this as a region seed. Seeds can be chosen at random or via a number of other initialization procedures. Folch and Spielman [19] have found that a purely random selection of seeds yields the most homogeneous regions and that approach is used here. Tracts contiguous to the seed are added one-by-one to the seed tract to build up the region. Once the set of tracts adjacent to the seed tract has been exhausted, the set of tracts eligible to join the region is expanded to include tracts contiguous to the tracts previously added. The strategy of building concentrically outward from the seed was adopted to ensure that the initialization produced compact regions as opposed to sinuous “gerrymanders.” Region construction stops and tracts are no longer added to the seed once the region satisfies all of the user-specified criteria (i.e., meeting or exceeding the CV and/or population thresholds). If a randomly selected seed meets all user-specified constraints then adding tracts to it is not necessary. A region can therefore be made up of one or more census tracts. Once that region is complete, another seed is chosen from the set of unassigned tracts, and the construction process repeats. This procedure iterates until no other feasible regions can be built. This typically results in a set of leftover census tracts. These leftover tracts are then added to existing regions, after verification that the newly expanded region still meets the user-specified constraints. A feasible solution is one in which each tract is assigned to a single region, and each region meets the CV and/or population constraints. Each run of this phase is very fast and can be repeated thousands of times. From this large set of feasible partitions the “best” partition is taken, where the best partition is the one with the most regions. In the case of a tie in the number of regions, we select the solution with the lowest *SSD*.

The second phase of the max-*p* algorithm swaps tracts between spatially adjacent regions in an effort to reduce the aggregate attribute heterogeneity within the regions, as measured by the sum of the squared deviations from the mean of the region (see equation 1). The max-*p* is a heuristic optimization algorithm; Folch and Spielman (2014) [4] show that using an internal-variance-minimizing objective function like SSD finds the minimum-variance partition of the input map over 95 percent of the time. Areas are swapped iteratively, and each iteration tries to identify the best of all feasible swaps of a single tract between regions. A feasible swap is one that does not change the number of regions identified in the first phase (regions cannot be created in the optimization phase), and one that ensures that all regions remain feasible after the swap. A tabu search strategy [20] is used to prevent backtracking to earlier solutions and to avoid getting trapped in suboptimal solutions. Stopping criteria prevent the algorithm from continuing to search once further improvement appears unlikely or some user specified maximum number of swaps occurs [4, 18].

In both the initialization and the optimization phases we rely on equations provided by the US Census Bureau in [21] to calculate the region-level CV for each input variable. The general approach to calculating region-level CV is to consider the standard errors of the input variables for each tract in a region. For derived proportions like average household income or percent Asian one has to consider the standard errors of both the numerator and the denominator. The procedure is fairly straightforward and well described in [21].

### Data

The algorithm accepts a set of ACS variables and their margins of error as input data. The ACS is widely used in the social sciences, and in an effort to illustrate the utility of our approach across a wide variety of social-scientific domains we construct four attribute scenarios: “general,” “poverty,” “transportation,” and “housing” (see Table 1). The data themselves come from the 2007–2011 ACS.

**Table 1:** Attribute and Scenario Summary.

	General	Poverty	Transportation	Housing
Average number of rooms	X			X
Average household income	X			
Persons per housing unit	X			
Percent occupied	X			X
Percent married	X			
Percent bachelor’s degree or higher	X			
Percent same housing unit last year	X			
Percent white	X			
Percent black	X			
Percent Hispanic	X			
Percent under 18	X			
Percent 65 and older	X			
Housing cost as share of income (owners)		X		
Housing cost as share of income (renters)		X		
Percent of children above poverty		X		
Percent of population above poverty		X		
Percent employed		X		
Vehicles per person			X	
Average commute time			X	
Percent drove alone			X	
Percent transit			X	
Average home value (owners)				X
Average rent				X
Percent owner occupied				X
Percent renter occupied				X
Percent single family housing unit				X

For the examples that follow, we constructed 18 data sets for each scenario, where each data set described a Metropolitan Statistical Area (MSA). We chose the 18 MSAs manually to represent both the range of US cities (population sizes and growth rates) and geographic variations within the United States (by selecting two cities from each of the nine US census divisions) (see Table 2).

**Table 2:** Metropolitan Statistical Areas Studied.

MSA	Census Division	2010 Population	2000–2010 Population Change
Atlanta GA	South Atlantic	5,268,860	24.0%
Austin TX	West South Central	1,716,289	37.3%
Birmingham AL	East South Central	1,128,047	7.2%
Boston MA	New England	4,552,402	3.7%
Buffalo NY	Middle Atlantic	1,135,509	−3.0%
Chicago IL	East North Central	9,461,105	4.0%
Cleveland OH	East North Central	2,077,240	−3.3%
Hartford CT	New England	1,212,381	5.6%
Kansas City MO	West North Central	2,035,334	10.9%
Los Angeles CA	Pacific	12,828,837	3.7%
Minneapolis MN	West North Central	3,279,833	10.5%
Nashville TN	East South Central	1,589,934	21.2%
Oklahoma City OK	West South Central	1,252,987	14.4%
Phoenix AZ	Mountain	4,192,887	28.9%
Pittsburgh PA	Middle Atlantic	2,356,285	−3.1%
Portland OR	Pacific	2,226,009	15.5%
Salt Lake City UT	Mountain	1,124,197	16.0%
Washington DC	South Atlantic	5,582,170	16.4%

Note: Population change measured using 2009 MSA definitions.

### Data Preparation

In addition to the substantive decisions on the goals, constraints, data, and algorithm discussed above, a number of practical decisions are needed to allow the approach to run smoothly. We remove from the analysis all tracts that do not have households. These tend to be uninhabited places such as large parks or bodies of water, or institutional locations such as large prisons or employment centers. This exclusion is necessary because we measure various ratios and proportions, and zero-household tracts or tracts with missing attributes would force a divide-by-zero operation that would derail the algorithm.

Another problematic issue is the MOE on attributes with a zero estimate. The approach used by the USCB to compute MOEs does not accommodate zero estimates, so all zero estimates in each state receive the same MOE [22]. For example, in the 2007–2011 data Ohio has 934 tracts with zero public transit commuters; each of these estimates has an MOE of ±89. In contrast, 57 tracts have only one transit commuter, but their MOEs range from just 2 to 5 (see 2007–2011 American Community Survey table B08134). Because of the high MOEs on zero estimates, we simply reset them to zero. While this assumes no uncertainty in the estimate, it is preferable to the high MOE from the published data.

Since the ranges of the input data are quite heterogeneous, e.g., dollars, number of rooms, percentages, etc., we standardize the input data using z-scores. There are multiple standardization procedures that could potentially be used. Tarpey [23] suggests that the optimal transformation for clustering applications is one in which the between-cluster variance is maximized, but Steinley [24] has argued that the choice of standardization procedure is unlikely to have an overall detrimental effect on classification performance. Because we compute the sum of squared deviations from the mean in our objective function, it is important that these deviations be on the same scale; otherwise a variable on, say, a dollar scale would have much more impact on the objective function than one on a ratio scale.

An additional challenge is potential redundant information in the input vectors. For example, in the transportation scenario we expect vehicles per person and percent who drove alone to be correlated. To account for this redundancy, principal components are calculated on the standardized data, and each of the resulting components is included in the analysis but is weighted according to the amount of variance it explains. This approach allows us to capture 100 percent of the variance in the input data while ensuring that correlated variables do not have a disproportionate impact on the objective function. The intention is to use all the information, but to give more weight to those components that contribute more to the overall variation in the data. One might be able to avoid the use of principal components by manually weighting variables, and this may make sense in certain applications. However, for the demonstration below, we decided to avoid such a subjective exercise.

When an estimate is very low, CVs tend to be extraordinarily high, as the Ohio transit commuter example illustrates. In places where the estimate for a particular variable is very low we remove the CV constraint—specifically, in the case of variables that are proportions where the estimate is less than 5%. For example, if the estimated percent African-American in a census tract was less that 5% the CV constraint for that variable would be removed because it would be very difficult to reduce the CV without building a very large region. Thus in some regions, for some variables, it is possible for the CV to exceed the user-specified threshold. This approach is based both on a pragmatic desire to prevent rare phenomena from dominating the classification and on a recommendation in [5], which states that a hard CV threshold “does not apply in some instances: specifically, for estimates of proportions that are less than 5 percent of a population group in an area. The formula for estimating the coefficient of variation is very unstable for estimates of small proportions, and the estimated coefficients can be misleadingly large” (pp. 67, 72). For example, exurban locations tend to have few transit options, so the CVs on the share of workers using transit tend to be quite high. If we do not ignore the CV, then the region would need to contain many tracts in order to meet the user-specified CV threshold.

Geographic irregularities can also confound the algorithm. Since a region must consist of spatially contiguous tracts, islands can make it hard to find feasible solutions. Tracts located on islands are not contiguous to the mainland and may not be able to form a region that meets user-specified targets because there is a limited number of tracts from which a region can be built and an island may not contain enough tracts to meet the user specified threshold. In the case of Lindo Isle and Balboa Island in the Los Angeles MSA, we create an artificial link to the mainland based on bridge locations. In contrast, we entirely exclude Grand Island in the Buffalo MSA, since it is on the edge of the MSA and is somewhat distinct from the more urban mainland communities. These are admittedly arbitrary decisions, but ones that an analyst familiar with an area can likely make on the basis of local context.

### Evaluation of Results

It is fairly simple to show that the regions produced by the algorithm achieve a user-specified uncertainty threshold; however, demonstrating that the resulting regions do not alter spatial patterns in the input data is a more difficult task. We have developed a suite of statistical and visual evaluation tools to allow a user to evaluate output from the algorithm objectively and subjectively. We use two statistical metrics to identify the amount of information retained from the regionalization. The first summary statistic is simply the number of tracts per region. Higher values mean that on average more tracts need to be grouped together to form feasible regions. This measure is useful to compare solutions across MSAs (which all have a different number of input tracts). This measure can also be useful in variable selection, one might be considering multiple poverty scenarios, each defined by a different ensemble of variables. Each of the poverty scenarios might yield a different average number of tracts per region. If the scenarios were substantively similar one might choose the set that yielded the smallest number of tracts per regions. The set of variables with the smallest number of tracts per region would maximize the geographic resolution of the output by minimizing the amount of aggregation necessary to meet constraints. When one must compare a set of possible solutions for a single MSA, this statistic can be reduced to the total number of regions.

The second metric (*S*_*j*_) attempts to quantify information loss through aggregation. This is measured by comparing the region-level estimates for each variable to the corresponding estimates for the tracts that constitute the region. The statistic *S*_*j*_ measures whether the region-level estimates for a given variable are within the margins of error of their constituent tracts. If a region-level estimate is within the margin of error of all its constituent tracts, then there is no information lost through aggregation; information loss increases as the 90 percent confidence intervals of more and more tract-level estimates do not overlap with the region’s estimate. Formally:

$$\begin{array}{c} \begin{array}{c} S_{j}=\frac{1}{n}\sum_{k}\underset{i\in k}{\Sigma }r_{kij},\;\text{where}\\ r_{kij}=\left\{\begin{array}{cc} 1 & \;\text{if}\;|a_{ij}-a_{kj}|<e_{ij}\\ 0 & \;\text{otherwise}. \end{array}\right. \end{array} \end{array}$$ (2)

For each tract *i* within each region *k*, we evaluate if the difference between the tract’s attribute value, *a*_*ij*_, and the region’s attribute value, *a*_*kj*_, is within the tract’s margin of error, *e*_*ij*_. The true cases are summed and divided by the total number of tracts (*n*). *S*_*j*_ therefore indicates the share of all tracts that are assigned to a region with no information loss for attribute *j*. A global version of *S*_*j*_ can be computed as the weighted average over all the attributes:

$$\begin{array}{c} S=\frac{1}{n*J}\sum_{k}\sum_{i\in k}\sum_{j}r_{kij}=\frac{1}{J}\sum_{j}S_{j}. \end{array}$$ (3)

*S* provides a single value for the overall success of the solution.

**Fig 2:**
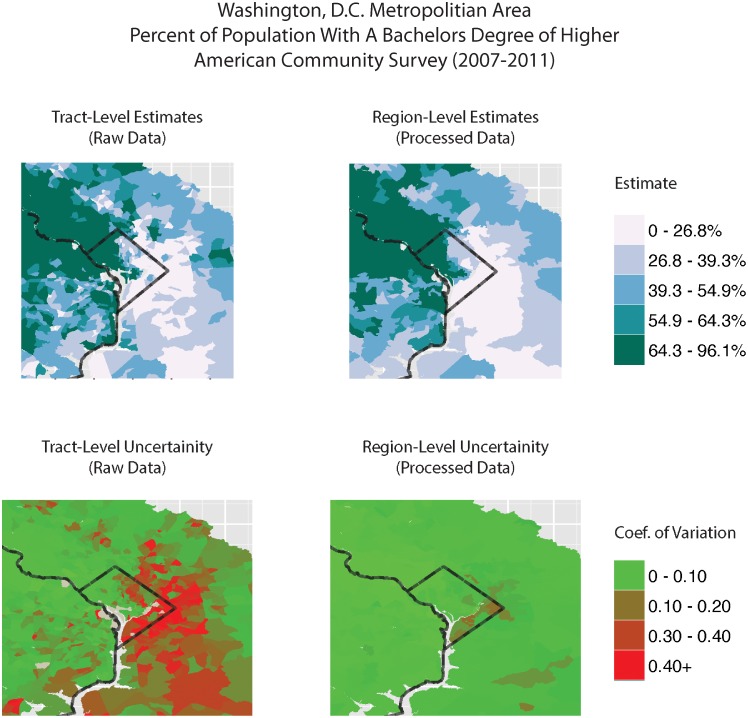
Maps of Regionalization input and outputs for Washington, DC.

**Fig 3:**
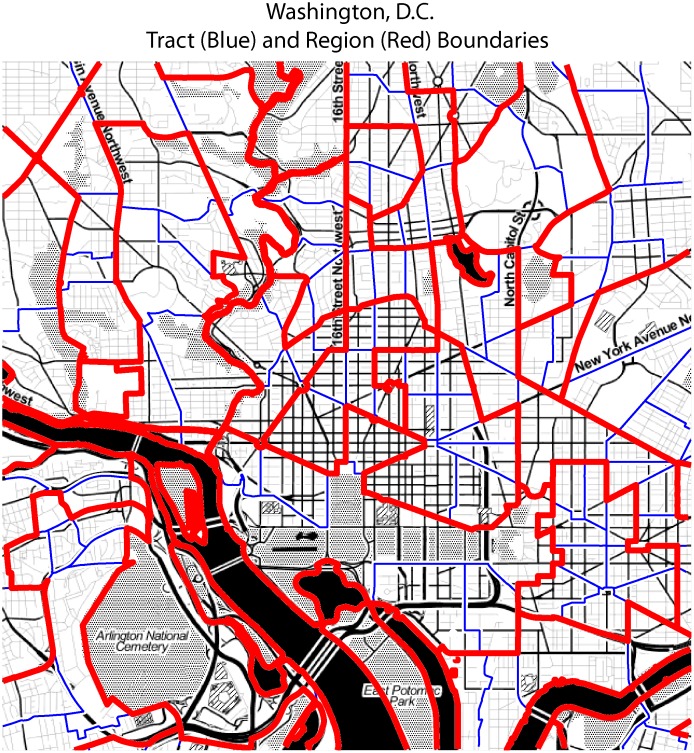
Region and tract boundaries in central Washington, DC. Background Image Source: Stamen Design/Open Street Map.

Visually, a user with local knowledge would find the maps of region boundaries and thematic maps an important evaluation tool. Fig. 2 shows the spatial pattern of estimates and CV for the percent of the population with a bachelor’s degree or higher at both the tract (input) and region (output) scales for Washington, DC. The top choropleth maps show estimates (using the same class breaks), and the lower row shows the CV for those estimates. Green regions of the lower maps have high-quality estimates; brown or red regions have poor estimates. Generally, the macrospatial pattern of higher educational attainment in the northwest and lower attainment in the southeast is preserved by the regionalization, but the CVs are markedly improved. A second visual evaluation tool is an examination of the region boundaries. Fig. 3 shows the results from the general scenario for a section of the city. A user with local knowledge could evaluate the coherence of the solution—that is, whether the regions seem like reasonable divisions of the city, or whether the regions mix different types of neighborhoods (as in lower right of Fig. 1).

**Fig 4:**
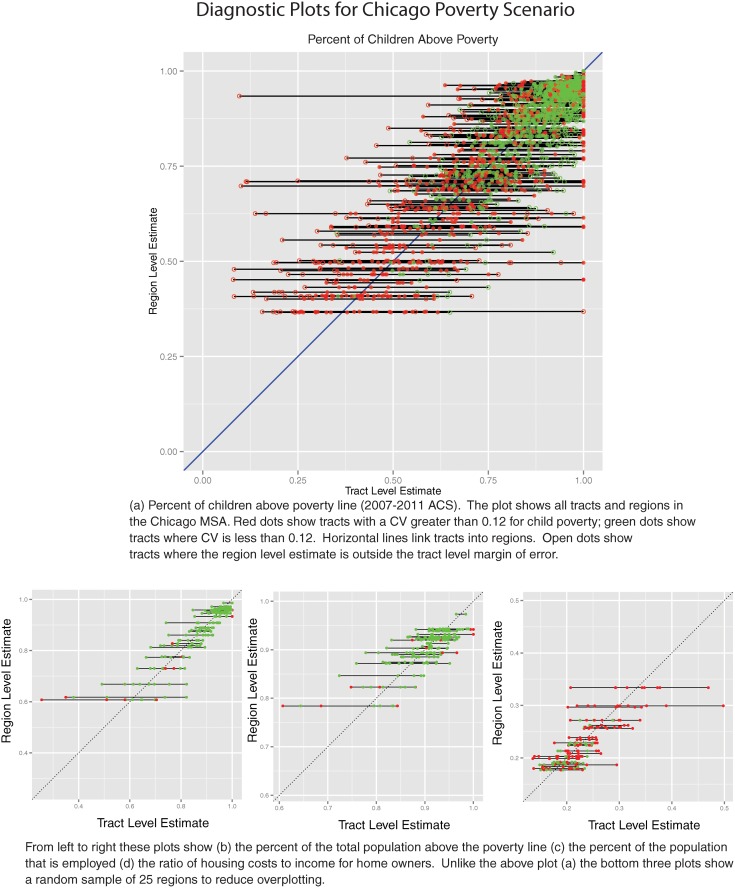
Chicago Diagnostic Plots.

Another visual strategy is to plot tract-level estimates and region-level estimates on a scatter plot, as in Fig. 4. Each point in the figure represents a tract. The position of the point in the graph is determined by the tract-level estimate from the ACS (x-axis) and the region estimate determined from the algorithm (y-axis). The color of the dot shows its initial condition: green points indicate tracts that meet or exceed the user-specified CV threshold, 0.12 in this case, and red points are tracts that need to be fixed by the algorithm. In the large graph in Fig. 4a, tracts where the tract-level margin of error includes the region-level estimate are depicted with a solid dot, and tracts where the region-level estimate is outside the tract-level MOE are shown with a hollow dot. The ratio of solid points to all points equals the *S*_*j*_ value for that attribute. This diagnostic plot does not work with count estimates (i.e., number of children under 5) because counts for groups of tracts (regions) will always be higher than counts for individual tracts. The horizontal bars link the constituent tracts of a region. Ideally, the horizontal bars would be short and centered on the 45-degree line, an indication that the region contains similar tracts and that the region- and tract-level estimates are similar. The axes of the plots are linked to the observed range of *tract*-level estimates; thus a plot like Fig. 4d, which shows the ratio of housing costs to income for homeowners, illustrates the fact that the range of observed values at the tract level is greater than the range of observed values at the region level. Similarly, there is more variance in the tract-level estimates than in the region-level estimates. The reduction in variance and compression of the range of observed values is illustrated by the lack of points above 0.35 on the y-axis. Aggregation necessarily reduces variance, but an ideal diagnostic plot would have points clustered along the diagonal *and* similar ranges for tract- and region-level estimates.

### Open Source Code, Data, and Results

The algorithm is fully open source, was developed in Python (regionalization) and R (evaluation), and is available free on GitHub (https://github.com/geoss/ACS_Regionalization). Additionally, all of the results described in the subsequent section and the code to reproduce all charts and figures are available on GitHub. The algorithm uses only open-source free software and relies heavily on the PySAL library [25]; maps and figures are produced in R using the ggplot2 library [26]. While the use of these tools requires some programming experience, the GitHub site includes a step-by-step tutorial that should allow users with minimal programming experience to use the methods outlined here. In addition shapefiles and input data for each of the scenarios for each of the MSAs have been posted to GitHub.

## Demonstration

Table 3 presents data from the poverty scenario for a selection of four adjacent census tracts from the Logan Square area of Chicago. The first row of the table shows estimates for housing cost as a share of income for home owners, a measure of housing affordability. From the estimates alone, tract 222800 appears to be the least affordable, with a rate of 63.3 percent; however, this tract also has an MOE of 114.5 percent, indicating that we are 90 percent certain that the true estimate is within the range of 0 to 177.8%, so this tract could actually be the highest or lowest for the percent of homeowner income spent on housing. The table also shows that high uncertainty on one attribute does not entail high uncertainty for all attributes for that tract. Tract 222800 has the highest CV for two of the attributes, but it has one of the lowest CVs for percent employed. Tract 222900 is the most consistent across attributes in terms of CV, but none of its attributes have CVs below the recommended threshold of 0.12, while the other three tracts all have at least one CV below the threshold.

**Table 3:** Estimates and Uncertainty for Selected Census Tracts, Poverty Scenario for Chicago MSA (Cook County).

	222600	222700	222800	222900
Housing cost as share of income (owners)				
Estimate	28.9%	28.4%	63.3%	46.5%
MOE	15.7%	10.2%	114.5%	19.7%
SE	9.6%	6.2%	69.6%	12.0%
CV	0.331	0.218	1.099	0.257
Housing cost as share of income (renters)				
Estimate	32%	38.8%	39.2%	48.3%
MOE	9.1%	15.4%	15.6%	23.1%
SE	5.5%	9.4%	9.5%	14.0%
CV	0.173	0.242	0.243	0.291
Children above poverty				
Estimate	89.7%	53%	33.7%	59.2%
MOE	19.5%	26.8%	29.9%	18.2%
SE	11.8%	16.3%	18.2%	11.1%
CV	0.132	0.308	0.54	0.187
Total above poverty				
Estimate	78.7%	74.7%	61.9%	64.6%
MOE	10.2%	7.5%	21%	14.8%
SE	6.2%	4.5%	12.8%	9.0%
CV	0.078	0.061	0.207	0.139
Percent employed				
Estimate	94.9%	78.5%	88.9%	87.7%
MOE	3.6%	8.5%	6.2%	31.0%
SE	2.2%	5.2%	3.8%	18.8%
CV	0.023	0.066	0.042	0.215

The challenges of measuring poverty in Chicago are not confined to these selected tracts. Only 78 of the 2,210 tracts in the MSA meet Citro and Kalton’s [5] recommended CV threshold of 0.12 on all five attributes. Table 4 shows the full distribution, and the more positive result that approximately 62 percent of the tracts meet the threshold for at least three of the five attributes. Table 5 shows that the pattern in Table 4 can be partially explained by variation in the overall quality of estimation of specific attributes—the attribute housing cost as share of income for renters, for example, meets the 0.12 CV threshold in only 194 tracts (8.8%).

**Table 4:** Number of Tracts Meeting Uncertainty Threshold (CV = 0.12), Poverty Scenario for Chicago MSA.

Number of Attributes	Number of Tracts
0	32
1	164
2	645
3	698
4	593
5	78
Total	2,210

**Table 5:** Number of Tracts Meeting Uncertainty Threshold (CV = 0.12), Poverty Scenario for Chicago MSA.

Attribute	Number of Tracts
Housing cost as share of income (owners)	800
Housing cost as share of income (renters)	194
Children above poverty	1,248
Total above poverty	2,056
Percent employed	2,012

With this diagnostic information in hand, one solution might be to collapse the owner and renter affordability estimates into one overall affordability measure. The weakness of this approach is that owning and renting housing are quite different. Owners and renters may differ in substantive ways other than tenure, and collapsing the variables might mask differences that are important from a policy perspective. For our purposes, we assume that keeping these two high-uncertainty attributes separate is advantageous. Similarly, we assume that all of the variables in the poverty scenario are necessary. Our aim here is more pedagogical than empirical, and a different set of variables would not substantively alter the illustration of the use of the algorithm that follows.

The regionalization algorithm produces 256 regions for the Chicago MSA, given the variables in Table 3, a maximum CV of 0.12, and no region-level population constraints. On average this is 8.6 tracts per region. The accuracy measure *S*_*j*_, which measures the share of tracts whose tract attribute value is “close” to the corresponding region attribute value, shows good results in general. *S*_*j*_ ranges from 0.758 for proportion of total population in poverty to 0.897 for housing cost as share of income (owners). The overall accuracy (*S*) is 0.836. These results are summarized graphically in Fig. 4. Ideally the horizontal bars linking tracts would be short and centered on the 45 degree line, an indication that the region contains similar tracts and that the region estimate and the tract estimates are similar. The large share of red in Fig. 4d (lower right) indicates that this variable is particularly uncertain at the tract scale, especially in contrast to the variable in Fig. 4b (lower left). In Fig. 4 wide bars are generally terminated by a red point, a tract with an uncertain estimate, suggesting that the estimate may not be as different from contiguous areas as the plot suggests.

### Variations in User Selections

In the previous example the number of attributes and the maximum CV value were fixed. In this section we look first at the effect of varying the maximum allowable uncertainty in the regionalization solution. Lower levels of uncertainty give more confidence in the estimates but require more aggregation of tracts leading to larger regions. To illustrate the impact of the user-specified CV threshold we rerun the Chicago scenario described above with five different maximum CV values (0.05, 0.10, 0.15, 0.20, and 0.40). Table 6 shows a dramatic reduction in the number of regions as the CV threshold decreases. When the maximum CV is set to 0.40, a level generally considered too high for research, there are on average 1.4 tracts per region. At the most restrictive level, CV = 0.05, there are approximately 43 tracts in the average region. When the CV threshold is set at 0.05 there is a significant loss of spatial resolution; the Chicago MSA is described by only 51 geographic zones. If one is willing to accept more uncertainty in the data there are significant gains in spatial granularity, at a CV threshold 0.40 the MSA is described by 1573 zones. The loss in attribute information is not as dramatic as the loss in spatial information. Even at the most stringent uncertainty level, over 78.5 percent of the tract estimates are located within the margin of error of their assigned region.

**Table 6:** Regionalization Results Summary, Variation in Maximum CV Value, Poverty Scenario for Chicago MSA.

Maximum CV	*S*	Number of Regions	Areas Per Region
0.05	0.785	51	43.196
0.10	0.823	193	11.415
0.15	0.846	393	5.606
0.20	0.875	639	3.448
0.40	0.925	1573	1.401

Next we consider the impact of changing the number of attributes. Again using the Chicago poverty scenario, we compute a regionalization solution for a single attribute, two attributes, and so on up to all five attributes. We hold the CV constant at 0.12 for these cases. For this example, attributes are added sequentially so that the variable with the lowest tract-level CV is added first, and then other variables are added until the worst performer is included. Table 7 shows the order in which variables are added and the accuracy rate by variable (*S*_*j*_) for each solution. Percent employed has a relatively low tract CV (see Table 5), which is reflected in a solution with an accuracy level of 0.991 and 2,021 regions (Table 8). As more attributes are included in the regionalization, the regions need to accommodate attributes with different spatial patterns in their CVs, and as a result the accuracy level (*S*_*j*_) for percent employed steadily declines, but still remains relatively high at 0.832. This decline in accuracy holds for all attributes as more attributes are added. Table 8 shows that the average *S*_*j*_ declines and region size grows as more attributes are added.

**Table 7:** Accuracy Results by Attribute (*S*_*j*_), Variation in Number of Attributes, Poverty Scenario for Chicago MSA.

	Number of Attributes				
	1	2	3	4	5
Percent employed	0.991	0.980	0.928	0.887	0.832
Total above poverty		0.968	0.887	0.845	0.758
Children above poverty			0.915	0.879	0.814
Housing cost as share of income (owners)				0.922	0.882
Housing cost as share of income (renters)					0.897

**Table 8:** Regionalization Results Summary, Variation in Number of Attributes, Poverty Scenario for Chicago MSA. Note that *S* is the average of the columns in Table 7

Number of Attributes	*S*	Number of Regions	Areas Per Region
1	0.991	2021	1.090
2	0.974	1950	1.130
3	0.910	1346	1.637
4	0.883	923	2.387
5	0.836	256	8.605

### Comparisons Across MSAs and Scenarios

**Fig 5:**
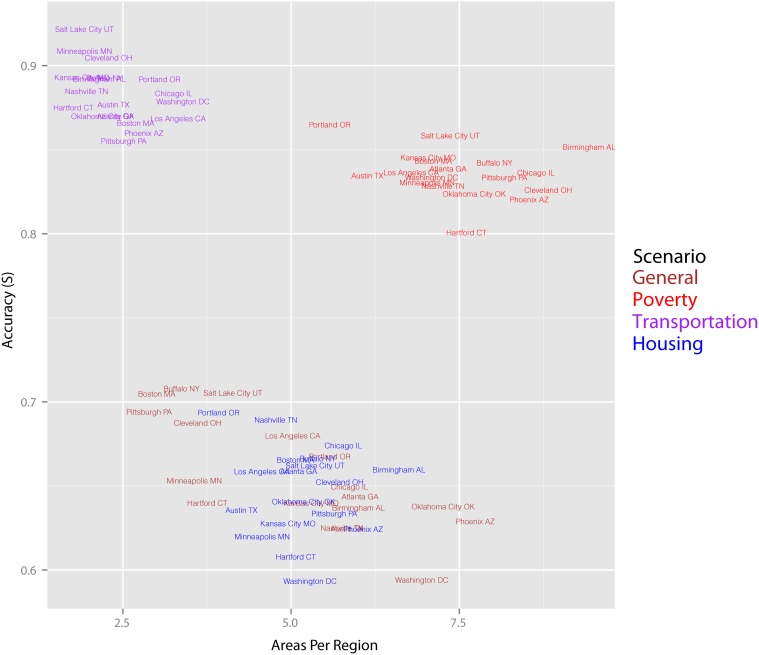
Regionalization Results Diagnostics, 18 MSAs and 4 Scenarios.

To provide perspective on variation by city types and attribute types, we applied the algorithm to 18 MSAs (Table 2) and four scenarios (Table 1), 72 total cases. In all cases we used a CV of 0.12 and no population constraints. Fig. 5 summarizes the results in terms of the two metrics: accuracy (*S*) on the y-axis and areas per region on the x-axis. What is clear from the plot is that differences in attribute bundle are more powerful in determining the general form of the solution than differences in MSA—all the results from a particular scenario are clustered together, while the results for a particular MSA are scattered around the plot.

This is not to say that MSA does not matter. A two-way ANOVA comparing the scenario-level and the MSA-level means of *S* and tracts per region rejects the null hypothesis that within scenario-level means are the same (significant at 0.001 level) and within scenario MSA-level means are the same (significant at 0.01 level

## Discussion

The regionalization method presented above is a Band-Aid for the ACS data. That is, it addresses an immediate problem (data quality) without getting at the root causes of the problem. The causes of the problems with the ACS data are complex and range from the statistical to the political. Given that systemic fixes are not likely to be forthcoming, we have tried to create a broadly applicable, intuitive, and usable method for post-processing the public-use ACS data.

The method we have presented is not ideal for all situations; in some cases abandoning existing census geographies may not be feasible. In these cases alternate methods, like Bayesian map smoothing [27], could be considered. Moreover, there are some problems with our approach that warrant discussion. We rely heavily on the point estimates in the construction of regions. First, without access to the raw surveys it is not possible to calculate the exact MOE for the new regions, the methods we use are the best available and recommended for use by the US Census Bureau. Second, the objective function does not account for the reliability of an estimate; it simply uses the published estimates for each variable selected by the user. While we do use the published MOEs to determine the feasibility of a region, the MOEs do not factor into the objective function. The algorithm is written in such a way that it is relatively easy to replace the objective function and the code is open source. One could create a new objective function that accounted for uncertainty in the input data however we were unable to identify one.

Each run of our algorithm will produce a different set of regions. Users of the algorithm must select a solution from a set of solutions that meet the user-specified constraints. In the preceding analyses we simply selected the solution with the lowest intraregion heterogeneity, but alternative criteria could have been used. For example, local knowledge could guide the selection: a user with an understanding of a metropolitan area might determine that one set of boundaries was a more coherent partition of the landscape. One could also try to select solutions that had desired geometric properties (such as compactness). A potentially fruitful area of future research is the application of methods like heuristic concentration [28], which attempt to identify an optimal solution utilizing multiple outputs from a heuristic optimization procedure.

From run-to-run the solutions are not entirely different, within a large set of potential solutions some tracts are often grouped together while other tracts often flip-flop between regions. These stable areas can be seen as “natural” regions, groups of tracts that share characteristics and the tracts that flip-flop between regions may be the edges of or transition zones between natural regions. We have tried to develop a statistical measure of the stability of region assignment. The stability of solutions from stochastically seeded algorithms is a long-running concern in the literature [29]. While it would be nice to know how stable a solution was, or which tracts tended to be grouped together, such knowledge would not substantively alter the application of the algorithm since all tracts must be assigned to a region.

Data-driven geographies of the sort created by the algorithm raise a more vexing issue. If geographies are designed around data, and the data change, should the geography change? A set of regions that works well for one release of the ACS might not achieve user-specified CV targets for the next release of the ACS. On the one hand, it seems sensible to design regions that maximize the utility of the data; on the other, it seems foolish to create ephemeral geographies that change from year to year. Moreover, having one set of regions for transportation and another set for housing-related problems may be problematic for certain uses. Using tracts as the building blocks of regions ameliorates these concerns to some extent, because tracts are relatively stable and therefore can always be recombined. For longitudinal comparisons regions created with one ACS release could be used to aggregate census tracts from prior (or later) releases of the ACS. This approach allows historical continuity but raises questions about the statistical optimality and substantive coherence of regions created using different data releases. In places with highly dynamic populations this concern may be more pronounced. However, these same concerns exist for the tracts themselves. If the “optimal” set of regions changes with a new release of the ACS data, it would be possible to retabulate the older data with the new regions. Census tract boundaries do change over time, however it is possible to account for these boundary changes using the tract relationship file published by the USCB concurrently with boundary changes.

## Conclusion

The American Community Survey, as the primary source of data about US neighborhoods, has important implications for social policy and social science. In their current form ACS data are unusable for many purposes. Unfortunately, the geographic hierarchy of census units has not evolved to match the reality of the new census ACS estimates. That is, tracts simply yield too few completed surveys to provide high-quality estimates, and counties (and cities, and even towns) are simply too large for many geographic and social-scientific questions. The Census Bureau recommendation that data users “combine geographic areas” can be seen as a statement about the suitability of the current census geographic system for many types of analysis. Unfortunately, this recommendation was accompanied neither by a set of guidelines for what constitutes a good aggregation nor by a set of tools to help users aggregate. New York City, manually, using local knowledge, made its own “Neighborhood Tabulation Areas,” which have a minimum population of 15,000 people [30]. Our algorithm accomplishes a similar end, and our diagnostics provide users some guidance in the aggregation process. The algorithm allows people to create bespoke geographic units of analysis. These custom divisions of space are a big conceptual change from the relatively static, general-purpose census tracts that have been in wide use for over 60 years, but we believe that this conceptual shift is necessary given the quality of the tract-level estimates published by the ACS. Using the algorithm requires a tradeoff that is not appropriate in all situations or for all audiences—one must be willing to reduce the number of geographic units of analysis. In the overwhelming majority of cases, however, this compromise does not sacrifice information.

However, reengineering geographic units opens a Pandora’s box of statistical issues. In the late 1970s [7] showed that it is possible to generate a perfectly negative (0.99) or a perfectly positive (0.99) correlation between the same variables in the same study region simply by changing the shape and scale of geographic units. Yule and Kendall (1950), quoted in [31], noticed a similar phenomenon and wondered whether the associations they observed in reaggregated data were “real” or “illusory.” These aggregation effects are a real concern and can be difficult to anticipate in statistical models [6]. However, the alternative to regionalization is using data that in many cases fail to meet even the most liberal standards of fitness for use.
